# Evolution and Divergence of H3N8 Equine Influenza Viruses Circulating in the United Kingdom from 2013 to 2015

**DOI:** 10.3390/pathogens6010006

**Published:** 2017-02-08

**Authors:** Adam Rash, Rachel Morton, Alana Woodward, Olivia Maes, John McCauley, Neil Bryant, Debra Elton

**Affiliations:** 1 Animal Health Trust, Lanwades Park, Kentford, Newmarket, CB8 7UU, UK; Rachel.Morton@aht.org.uk (R.M.); Alana.Woodward@aht.org.uk (A.W.); Olivia.Maes@aht.org.uk (O.M.); Neil.Bryant@aht.org.uk (N.B.); Debra.Elton@aht.org.uk (D.E.); 2 Crick Worldwide Influenza Centre, The Francis Crick Institute, 1 Midland Road, London, NW1 1AT, UK; John.McCauley@crick.ac.uk

**Keywords:** equine influenza, H3N8, haemagglutinin, neuraminidase, surveillance, United Kingdom

## Abstract

Equine influenza viruses (EIV) are a major cause of acute respiratory disease in horses worldwide and occasionally also affect vaccinated animals. Like other influenza A viruses, they undergo antigenic drift, highlighting the importance of both surveillance and virus characterisation in order for vaccine strains to be kept up to date. The aim of the work reported here was to monitor the genetic and antigenic changes occurring in EIV circulating in the UK from 2013 to 2015 and to identify any evidence of vaccine breakdown in the field. Virus isolation, reverse transcription polymerase chain reaction (RT-PCR) and sequencing were performed on EIV-positive nasopharyngeal swab samples submitted to the Diagnostic Laboratory Services at the Animal Health Trust (AHT). Phylogenetic analyses were completed for the haemagglutinin-1 (HA1) and neuraminidase (NA) genes using PhyML and amino acid sequences compared against the current World Organisation for Animal Health (OIE)-recommended Florida clade 2 vaccine strain. Substitutions between the new isolates and the vaccine strain were mapped onto the three-dimensional structure protein structures using PyMol. Antigenic analyses were carried out by haemagglutination inhibition assay using a panel of post-infection ferret antisera. Sixty-nine outbreaks of equine influenza in the UK were reported by the AHT between January 2013 and December 2015. Forty-seven viruses were successfully isolated in eggs from 41 of the outbreaks. Only three cases of vaccine breakdown were identified and in each case the vaccine used contained a virus antigen not currently recommended for equine influenza vaccines. Nucleotide sequencing of the HA and NA genes revealed that all of the viruses belonged to the Florida clade 2 sub-lineage of H3N8 EIV. Phylogenetic and sequence analyses showed that the two sub-populations, previously identified within clade 2, continued to circulate and had accrued further amino acid substitutions. Antigenic characterisation using post-infection ferret antisera in haemagglutination inhibition assays however, failed to detect any marked antigenic differences between the isolates. These findings show that Florida clade 2 EIV continue to circulate in the UK and support the current OIE recommendation to include an example of Florida clade 2 in vaccines.

## 1. Introduction

Equine influenza virus (EIV) is a major cause of acute respiratory disease—of a highly contagious nature—in horses and other equids. It is endemic in most parts of the world and can cause severe disruption to the racing and breeding industries. Like other influenza A viruses, EIV has two surface glycoproteins, haemagglutinin (HA) and neuraminidase (NA), which perform essential roles in virus entry and exit. HA is responsible for binding to host cell sialic acid receptors and promotes membrane fusion [1,2]. NA has sialidase activity, which is important for virus exit and release, but has also been suggested to have an early role in virus entry through the removal of decoy receptors in the host’s respiratory tract [3]. HA is a major target for neutralising antibodies and so is an important component of commercial vaccines. The importance of EIV NA to immunity however, is currently unknown, although antibodies to human influenza NA have been shown to contribute to protection [4]. NA has been the target of second-generation antiviral drugs to inhibit its function and prevent virus propagation. Although not routinely used in horses, NA inhibitors have been shown to have potential for the treatment of EIV infection [5,6].

H3N8 EIV was first isolated in 1963 during a widespread outbreak in the United States and has continued to circulate ever since [7,8,9]. It has been responsible for causing major outbreaks of disease among horses around the world, including in the UK in 1979, 1989 and 2003 as well as in South Africa in 2003, Japan and Australia in 2007, India in 2008–2009 and, more recently, in South America in 2012 [10,11,12,13,14,15,16,17,18]. Thought to have originated from an avian source in South America, the virus has since adapted to the horse and diverged into antigenically and genetically distinct lineages and sub-lineages [8,19,20]. The American and Eurasian lineages emerged during the 1980s and since then the American lineage has further evolved into the Kentucky, South American and Florida sub-lineages [20]. In the early 2000s, the Florida sub-lineage diverged into clades 1 (FC1) and 2 (FC2), and has been the dominant circulating sub-lineage ever since. Although not fully restricted geographically, FC2 viruses have predominately been isolated in Europe and Asia, whilst the majority of FC1 viruses have been isolated in North America [8,9,15,21,22,23,24,25,26].

Since being made compulsory in the racing industry in the early 1980s, vaccination has been widely used for the control of EIV. However, like other influenza A viruses, EIV undergoes antigenic drift through the accumulation of amino acid substitutions over time in HA, which has led to vaccine breakdown in the past [13,27,28]. EIV vaccines therefore need to be updated periodically and a formal process of vaccine strain selection, overseen by the World Organisation for Animal Health (OIE) has been in place for several years. An expert surveillance panel (ESP) makes vaccine strain recommendations based on genetic, antigenic and epidemiological data that are collected and reported to the panel on an annual basis [29]. Current recommendations are to include both FC1 and FC2 strains in vaccines [30]. Surveillance data are therefore crucial to this process. Our aim was to continue to monitor the genetic and antigenic changes occurring in EIV circulating in the UK. Here we present the HA and NA sequences, as well as antigenic characterisation of viruses isolated between 2013 and 2015 from disease outbreaks in the UK. We show that FC2 viruses have continued to diverge from the OIE-recommended vaccine strain into two sub-groups, with multiple amino acid substitutions occurring in both HA and NA.

## 2. Results

Between January 2013 and December 2015, 69 outbreaks of equine influenza in the UK were reported by the Animal Health Trust (AHT). These affected 28 counties in England, 8 in Scotland and 2 in Wales, with multiple outbreaks in some areas. Diagnoses were made by either qRT-PCR or nucleoprotein (NP)-ELISA performed on extracts from nasopharyngeal swabs submitted to the Diagnostic Laboratory Services at the AHT. In total, 84 samples from the 69 outbreaks tested positive. Virus was successfully isolated in embryonated hens’ eggs from 47 positive samples corresponding to 41 outbreaks. Apart from three isolates, all of the viruses were isolated from samples taken from either unvaccinated animals (*n* = 45), or animals whose vaccination status was out of date (*n* = 2). Ayrshire/1/13 and Ayrshire/2/13 were isolated from horses that had received a booster vaccination with ProteqFlu TE, a Canarypox vaccine expressing the HA from Ohio/03 and Newmarket/2/93, 6 months previously. These horses had both received the primary course of vaccinations 5–7 months before the booster. Buckinghamshire/1/14 was isolated from a horse that had a complete vaccination history and had received the last annual booster vaccination with ProteqFlu eight months previously, although this horse had received a primary course and previous booster vaccinations with Duvaxyn IE-T/IE-T Plus containing Suffolk/89, Newmarket/1/93 and Prague/56. However, the antibody titres of these vaccinated animals were unknown at the time of infection. Details of the virus isolates are summarised in Table 1.

### 2.1. Genetic Analyses—HA

**Figure 1:**
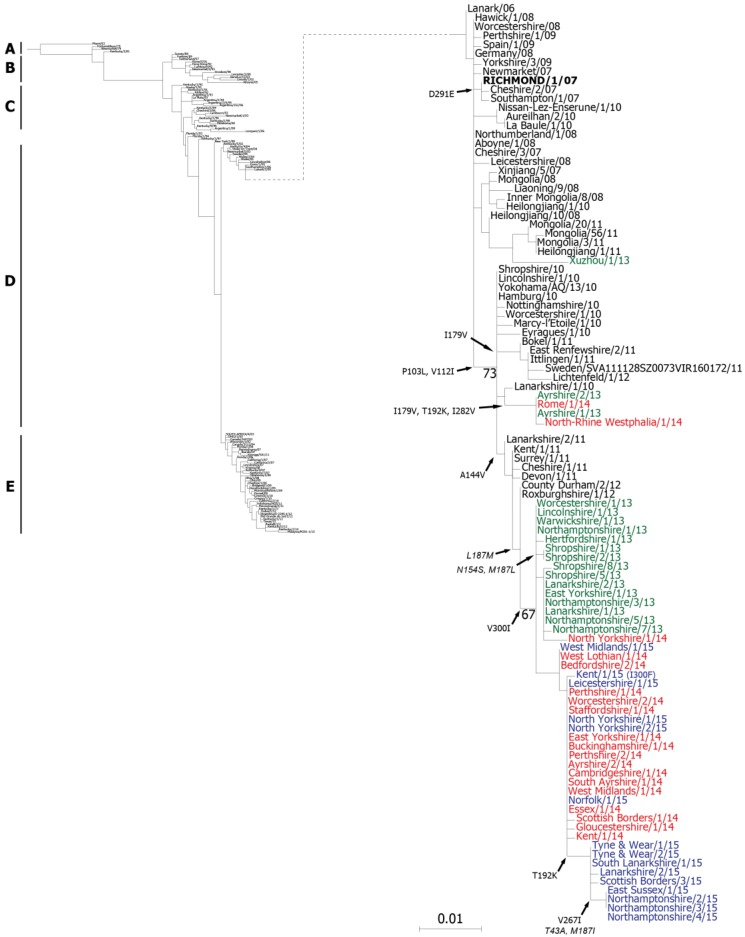
HA1 phylogenetic tree. Phylogenetic analysis of HA1 nucleotide sequences encoded by EIV. A maximum likelihood tree was created using PhyML version 3. Bootstrap values obtained after 100 replicates are shown at major nodes. Phylogenetic groups are shown by continuous bars on the left, **A**—pre-divergent, **B**—Eurasian, **C**—American (Kentucky and Argentinian), **D**—Florida sub-lineage clade 2, **E**—Florida sub-lineage clade 1. The Florida sub-lineage clade 2 group (**D**) has been enlarged and positioned to the right with a dashed line indicating its branch position in the tree on the left. Amino acid substitutions within clade 2 of the Florida sub-lineage are indicated at branch points or in brackets—HA1 substitutions are in normal text, HA2 substitutions are italicised. Sequences are coloured by outbreak date for the years 2013 (green), 2014 (red) and 2015 (blue) with older strains in black. The Florida clade 2 World Organisation for Animal Health (OIE)-recommended vaccine strain A/eq/Richmond/1/07 is shown in bold capitals. The HA1 sequences for the non-UK strains Xuzhou/1/13, Rome/1/14 and North-Rhine Westphalia/1/14 were obtained from Genbank (accession numbers KF806985.1, KR534268.1 and KJ538149.1 respectively).

The nucleotide sequence encoding HA was determined for each of the virus isolates by Sanger dideoxynucleotide sequencing and the resulting sequences were deposited in the GISAID EpiFlu™ database [31] (Table 1). For phylogenetic analyses, nucleotide sequences from strains representative of the different H3N8 EIV lineages were retrieved from the Genbank and GISAID databases. Due to a lack of available full-length HA sequences, a maximum likelihood phylogenetic tree was constructed using the nucleotide sequences for HA1 only (Figure 1). Distinct clusters within the phylogenetic tree corresponded to the pre-divergent, Eurasian and American lineages, as well as the two clades of the Florida sub-lineage. All of the sequences from the 2013–2015 isolates were located within the FC2 group, in the expanded section of the tree, however two distinct sub-groups were evident separating strains encoding the A144V substitution (144-group) from those with the I179V substitution (179-group; HA1 residues are numbered from the serine residue downstream of the predicted signal peptide). Only two of the isolates from the UK, Ayrshire/1/13 and Ayrshire/2/13 were within the I179V cluster, together with German (North-Rhine Westphalia/1/14) and Italian (Rome/1/14) strains isolated in 2014. The remaining UK isolates from 2013–2015 were grouped with strains isolated in previous years that encoded the A144V substitution (Figure 1). The more recent 2015 isolates formed a distinct group from the 2014 and early 2015 isolates (Figure 1). A Chinese isolate from 2013, Xuzhou/1/13 (Genbank accession number KF806985.1), clustered with other Asian strains from 2011, suggesting that another sub-group of FC2 may be circulating in Asia (Figure 1).

**Figure 2:**
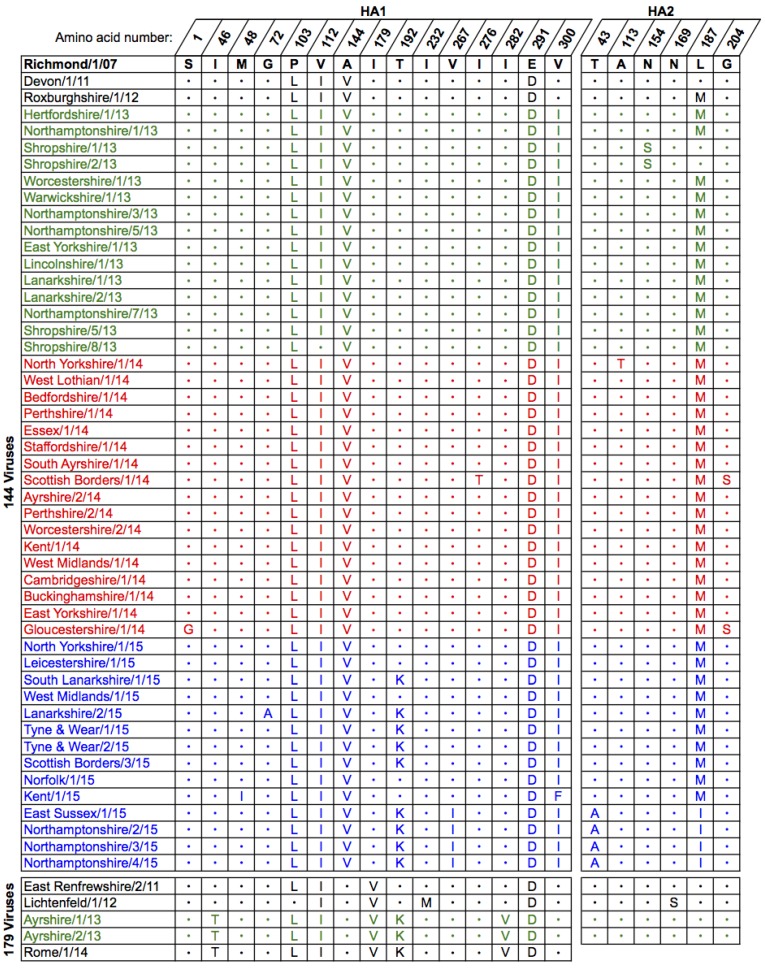
Amino acid substitutions within haemagglutinin (HA) between the OIE-recommended Florida clade 2 vaccine strain A/eq/Richmond/1/07 and strains isolated in the UK between 2013 and 2015. Isolates are grouped as belonging to either the 144- or 179-group, ordered by outbreak date and coloured by year: 2013—green, 2014—red, 2015—blue. HA1 residues are numbered from the serine residue downstream of the predicted signal peptide. Amino acid identity to A/eq/Richmond/1/07 is shown with a dot. Examples of strains from 2011 to 2012 are included to allow comparison with Woodward et al. (2014) [9]. The HA1 sequence for A/eq/Rome/1/14 was obtained from Genbank (accession number KR534268.1) and is included for comparison.

The derived amino acid sequences for the full-length HA from the 2013–2015 UK isolates were compared to the current OIE-recommended FC2 vaccine strain A/eq/Richmond/1/07 and representative UK isolates from 2011 to 2012 (Figure 2). As in the phylogenetic tree (Figure 1) the sequences divided into two separate groups, one containing the strains encoding the A144V substitution and the other containing those with the I179V substitution (Figure 2). All of the isolates encoded the amino acid changes P103L, V112I (except Shropshire/8/13) and E291D in HA1 compared to Richmond/1/07. The majority of the 144-group also encoded V300I in HA1 and L187M in HA2 (Figure 2). Of the 2015 isolates, 9 out of 14 encoded an additional substitution, T192K, within antigenic site B located at the top of the HA trimer. The most recent of these isolates contained a further three substitutions: V267I in HA1 and T43A and L187I in HA2 (Figure 2). These were the four isolates that formed the distinct group in the phylogenetic tree (Figure 1). The two 179-group isolates, Ayrshire/1/13 and Ayrshire/2/13, shared the same additional amino acid substitutions in HA1 as North-Rhine Westphalia/1/14 and Rome/1/14: I46T, (I179V), T192K and I282V (Figure 2). Unlike the 144-group, there were no substitutions within HA2 of the Ayrshire/13 viruses compared to Richmond/1/07 (Figure 2). None of the amino acid substitutions observed in either group affected glycosylation sites within HA when compared to the recommended vaccine strain. The genetic similarities between the two sub-groups and the recommended vaccine strain are shown in Table S1.

**Figure 3:**
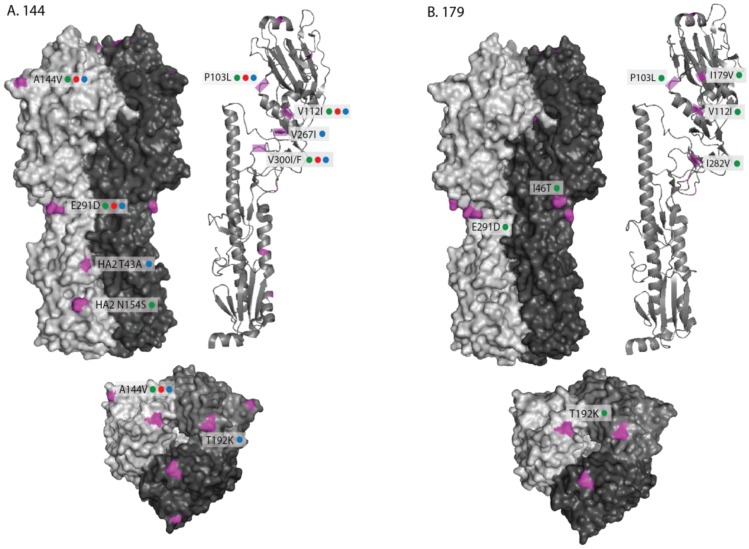
Structure of A/eq/Richmond/1/07 H3 HA [32] (RCSB Protein Data Bank, accession number- 4UO0) showing changes between 2013 (green dot), 2014 (red dot) and 2015 (blue dot) isolates from the 144 (**A**) and 179 (**B**) sub-groups and the OIE-recommended vaccine strain A/Eq/Richmond/1/07. Three views of HA structure are shown—side view, top view and a monomeric ribbon structure highlighting internal changes which are not visible on the trimeric structure. Changes are shown in purple and are labelled with amino acid substitution. The 144-group HA2 changes L187M/I (2013–2015) and G204S (2014 only) could not be mapped.

The locations of the individual amino acid substitutions for each sub-group (144 and 179) were mapped on the three-dimensional structure of the equine H3 HA (Figure 3A,B) [32] compared against the OIE-recommended vaccine strain A/eq/Richmond/1/07. For the 144-group, two changes within antigenic sites (A144V—site A and T192K—site B) were visible on the globular head of HA1 (Figure 3A). The E291D substitution was at the base of the globular head of HA1, whilst the two HA2 substitutions T43A and N154S were visible on the surface of the stalk structure (Figure 3). Four of the HA1 substitutions mapped to internal regions of the structure (P103L, V112I, V267I and V300I/F) and are shown on a monomeric ribbon structure (Figure 3). Two further substitutions residing at the C-terminal domain of HA2, L187M/I and G204S, could not be mapped as they were not present in the crystal structure.

The amino acid substitutions observed in the 179-group were also mapped on the equine HA structure (Figure 3B). In addition to the changes that were common to both the 144- and 179-groups when compared to A/eq/Richmond/1/07, one substitution (I46T) was visible on the surface at the base of the globular head of HA1 and two substitutions (I179V and I282V) mapped to internal regions of the structure and are shown on a monomeric ribbon structure (Figure 3).

### 2.2. Genetic Analyses—NA

**Figure 4:**
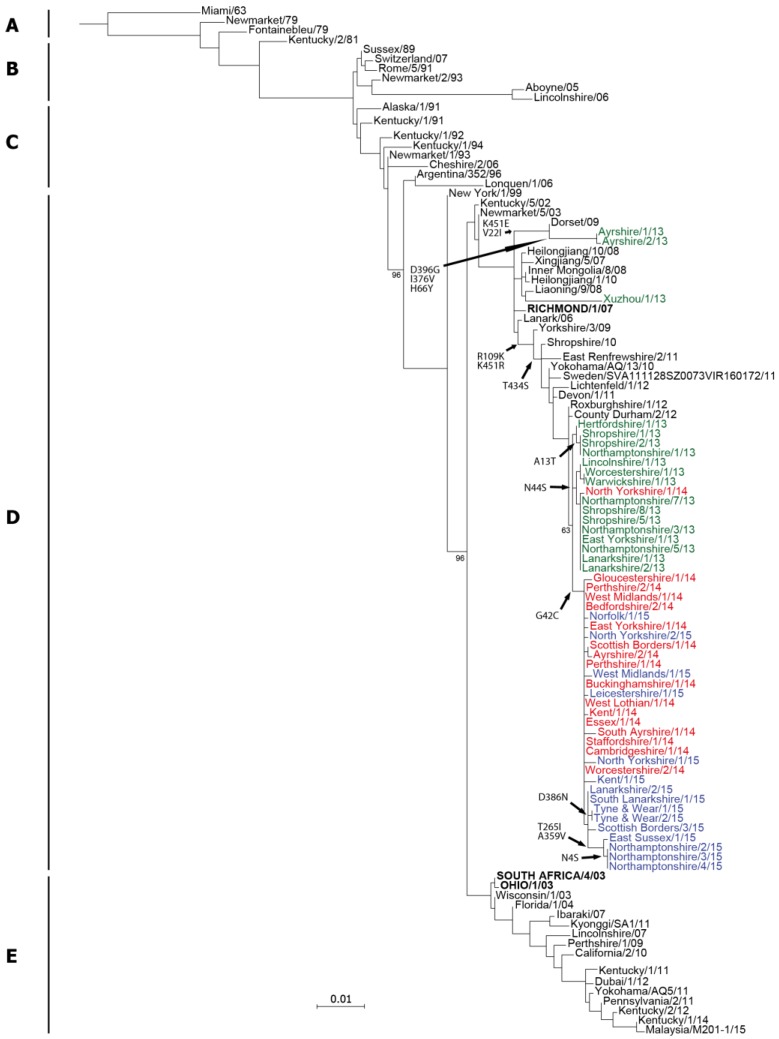
Phylogenetic tree of neuraminidase (NA) nucleotide sequences encoded by EIV. A maximum likelihood tree was generated using PhyML version 3, and phylogenetic groups are shown: **A**—pre-divergent, **B**—Eurasian, **C**—American (Kentucky and Argentinian), **D**—Florida clade 2, **E**—Florida clade 1. Bootstrap values are shown at major nodes. Amino acid substitutions within FC2 are indicated at branch points. Sequences are coloured by outbreak date for the years 2013 (green), 2014 (red) and 2015 (blue) with older strains in black. The FC2 OIE-recommended vaccine strain A/eq/Richmond/1/07 is shown in bold capitals.

As for HA, the nucleotide sequence encoding NA was determined for each of the virus isolates and deposited in the GISAID EpiFlu™ database [31] (Table 1). A maximum likelihood phylogenetic tree was constructed using NA sequences downloaded from the Genbank and GISAID databases (Figure 4). The tree topology was similar to the HA tree and all of the 2013–2015 isolates reported here clustered within FC2 (Figure 4). The 2013–2015 isolates also grouped in a similar pattern to the HA tree, with the most recent 2015 isolates forming a distinct sub-group (Figure 4). The Ayrshire/13 179-group viruses were positioned separately to the other 2013–2015 isolates and were most similar to the NA sequence from the 2009 UK isolate Dorset/09 (Figure 4).

**Figure 5:**
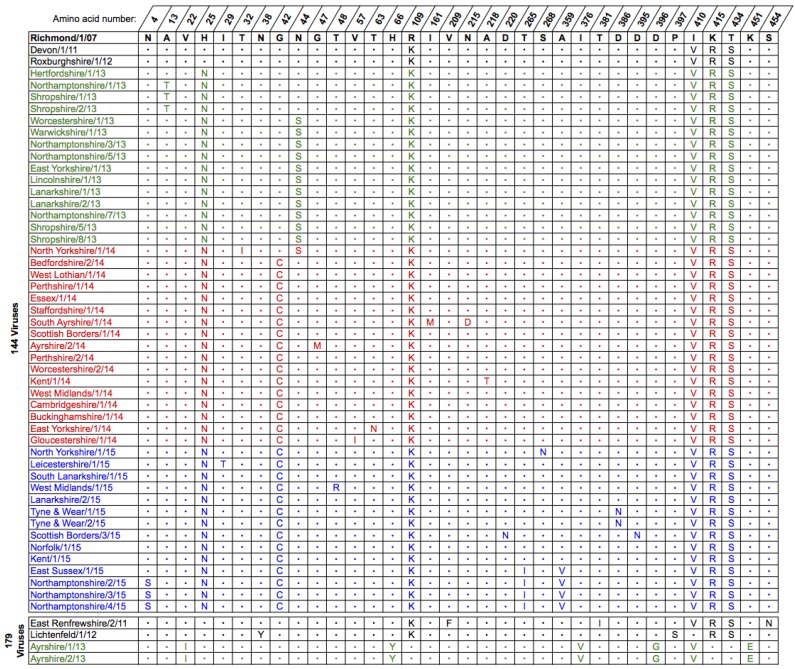
Amino acid substitutions within NA between the OIE-recommended Florida sub-lineage clade 2 vaccine strain A/eq/Richmond/1/07 and strains isolated in the UK between 2013 and 2015. Isolates are grouped as belonging to either the 144 or 179 HA1 group, are ordered by outbreak date and coloured by year as in Figure 1, Figure 2 and Figure 4: 2013—green, 2014—red, 2015—blue. Residues are numbered from the methionine residue at the start of the predicted signal peptide and have not been adjusted to correspond to N1 or N2 numbering. Amino acid identity to A/eq/Richmond/1/07 is shown with a dot. Examples of strains from 2011 to 2012 are included to allow comparison with Woodward et al. (2014) [9].

The derived amino acid sequences for NA from the 2013–2015 UK isolates were compared to the current OIE-recommended vaccine strain A/eq/Richmond/1/07 and representative UK isolates from 2011 and 2012 (Figure 5). As expected from the tree (Figure 4), and like the HA sequences (Figure 2), the sequences divided into two separate groups based on the 144 and 179 substitutions observed in HA. There were five amino acid substitutions common to all of the 144-group viruses: H25N, R109K, I410V, R415R and K434S (Figure 5). Isolates from late 2013, as well as the first isolate from 2014 (North Yorkshire/1/14), shared a substitution at position 44 (N44S), however this was not present in the other 2014 or 2015 isolates and appeared to be replaced by viruses encoding the G42C substitution (Figure 5). The four latest isolates from 2015, which formed a sub-group in the tree (Figure 4), had two further substitutions: T265I and A359V (Figure 5). The Ayrshire/13 isolates shared the I410V substitution with the 144-group and the 179 virus East Renfrewshire/2/11, but did not share any of the other substitutions. These viruses had five distinct substitutions compared to Richmond/1/07 and the other viruses: V22I, H66Y, I376V, D396G and K451E (Figure 5). None of the amino acid substitutions observed in the NA sequences affected glycosylation sites when compared to the recommended vaccine strain. In addition, none of the residues which form the active site or are involved in the catalytic function of the neuraminidase were affected. The H275Y mutation responsible for resistance to the antiviral drug oseltamivir was also not present in these viruses. The genetic similarities between the two sub-groups and the recommended vaccine strain are shown in Table S1.

**Figure 6:**
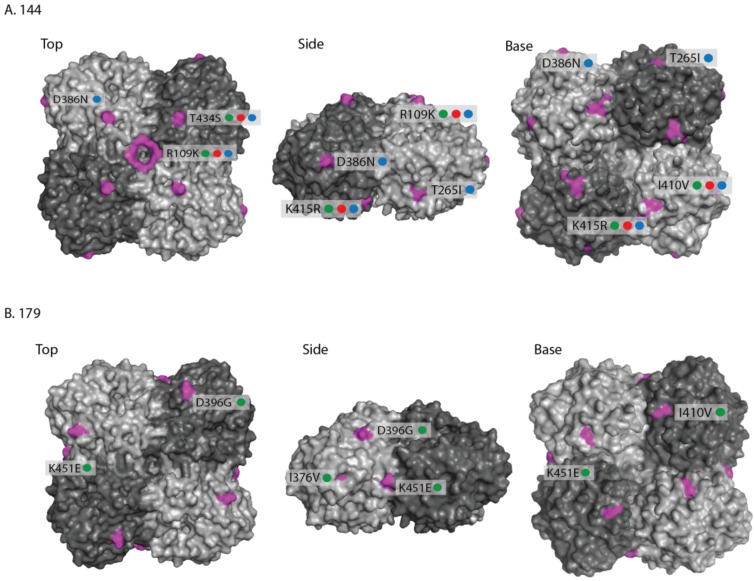
Structure of the neuraminidase (NA) protein [33] (RCSB Protein Data Bank, accession number- 2HTY) showing changes between 2013 (green dot), 2014 (red dot) and 2015 (blue dot) isolates from the 144- and 179-sub-groups and the OIE-recommended vaccine strain A/eq/Richmond/1/07. Residues are numbered according to the N8 sequence but shown on the structure of N1 [33]. Top, side and base views of the neuraminidase tetramer are shown for each sub-group. The 144-group change A359V is not shown, and N4S, A13T, H25N, G42C and N44S changes could not be mapped. 179-group changes V22I and H66Y could not be mapped.

In order to map the amino acid substitutions onto the three-dimensional structure of NA, the amino acid numbering of the protein was adjusted to correspond to the H5N1 NA protein structure database file 2HTY (Figure S1) [33]. Substitutions within the membrane anchor and stalk regions could not be mapped as these are not included in the solved protein structure. Of the 144-group substitutions, R109K and T434S were visible on the top of the globular head with the 109 substitution forming a ring at the centre of the tetramer (Figure 6). The T265I and D386N changes were visible on the side of the molecule, whilst the I410V and K415R mapped to the base of the globular head and were adjacent to one another (Figure 6). The A359V substitution observed in the most recent 2015 isolates did not map on the surface of the structure. Of the 179-group changes, the D396G substitution mapped to the top of the molecule, I376V and K451E to the side and I410V to the base of the tetramer (Figure 6).

### 2.3. Antigenic Characterisation

Isolates from each of the years reported here, and representative of each of the HA sequences, were characterised by haemagglutination inhibition (HI) assay against a panel of post-infection ferret antisera raised against representative strains from the Eurasian and American lineages (including the Kentucky and FC1 and FC2 sub-lineages). The panel included both of the OIE-recommended vaccine strains (Richmond/1/07 and South Africa/4/03) and antisera raised against viruses with 144 and 179 substitutions. The geometric mean titres (GMT) of duplicate assays are shown in Table 2. The titres achieved using the antiserum raised to the Eurasian lineage virus Newmarket/2/93 against the 2013–2015 isolates were the lowest, with titres to most strains 8- to 32-fold lower than the homologous virus, supporting the ESP recommendation in 2010 to replace Eurasian lineage strains in vaccines with a Florida clade 2 strain (Table 2) [34]. For all of the 2013–2015 isolates the highest titres were observed with either the Richmond/1/07 antiserum or the Ayrshire/1/13 antiserum, or both, ranging from 362 to 1024 (Table 2). The highest titres against both of the 179-group viruses, Ayrshire/1/13 and Ayrshire/2/13, were achieved using the homologous antiserum raised against the Ayrshire/1/13 isolate, although the Richmond/1/07 antiserum also reached a titre of 724 against Ayrshire/2/13 (Table 2). Titres against the 2013–2015 isolates were between 4- and 16-fold lower with the antiserum raised against the Florida clade 1 OIE-recommended vaccine strain South Africa/4/03, compared to the Florida clade 2 OIE-recommended vaccine strain Richmond/1/07 (Table 2), also supporting the 2010 ESP recommendation to include examples of both clades of the Florida sub-lineage in vaccines [34]. There were no obvious antigenic differences detected between the 2013–2015 isolates by HI assay using this panel of ferret antisera.

## 3. Discussion

Since its emergence in 1963, H3N8 equine influenza has diverged into two distinct lineages and three sub-lineages, with Florida clades 1 and 2 predominating in recent years. The current recommendation to include a representative from both FC1 and FC2 in vaccines was first made by the OIE in 2010 [34]. Examples of both clades have previously been isolated from horses in the UK, however only FC2 has been detected in the UK since 2010 [9,35].

The majority of EIV-positive samples submitted to the equine influenza surveillance programme at the AHT were from either unvaccinated horses or those that did not have up-to-date vaccination records. Only three cases of EIV infection in vaccinated horses were detected over the three years and in each case the vaccines used did not comply with the OIE recommendation to contain an FC2 strain.

Analysis of virus isolates from 2013 to 2015 revealed that, as in previous years, clade 2 viruses continued to circulate in the UK, whilst no clade 1 viruses were isolated during this time. Sequence analysis split the isolates into two sub-groups of clade 2, 144-group and 179-group, as reported previously, for both the HA and NA sequences [9]. Whilst the majority of UK isolates belonged to the 144-group, sequences published on Genbank from Germany and Italy showed that viruses belonging to the 179-group were also circulating in Europe during this time. The HA and NA sequences of the two sub-groups had diverged further, with 7 amino acid substitutions in HA and 12 in NA between the most recent viruses. Interestingly, the T192K substitution in antigenic site B observed in the 2013 179-group viruses was also present in more recent 144-group isolates from 2015. This would also suggest that the T192K substitution in the 144-group occurred independently from the same change in the 179-group. Despite two changes in antigenic sites, antigenic analysis using post-infection ferret antisera did not reveal any marked difference between the two groups. However, a weakness of the HI assay is that only substitutions appearing close to the receptor binding site are likely to interrupt antibody-binding activity that block binding of HA1 to sialic acid receptors on red blood cells. Other substitutions, which may alter binding of neutralising antibodies at other regions of the HA, for example antibodies that bind to the stem region of the HA [36,37], would not necessarily be detected using this assay. Although not in the stem region, the A144V substitution has previously been shown to affect neutralisation titres of post-infection equine antisera raised against an Argentinian lineage vaccine strain (La Plata/93) in an egg-based virus-neutralisation (VN) assay [38]. Moreover, horses vaccinated with the Argentinian lineage virus had increased temperatures and shed more virus following challenge with an A144V virus compared to horses vaccinated with a clade 2 virus lacking the A144V substitution [39]. Whilst the HI titres against the A144V virus were similar between the vaccine groups, the VN titres were reported to be in the order of 8-fold lower [39]. This suggests that the A144V substitution has an antigenic effect, which is not detected by post-infection ferret antisera, or indeed equine antisera in the HI assay.

Analysis of the NA sequences from the 2013–2015 isolates also showed divergence between the 144- and 179-group viruses, with at least 10 amino acid differences between the two sub-groups. The antigenic effects of these substitutions in NA are currently unknown and further work is needed to determine the role of antibodies to NA in the horse.

The continuing divergence of clade 2 viruses in Europe into the 144- and 179-groups may lead to the circulation of two antigenically distinct sub-lineages. A third group of clade 2 viruses appears to be circulating in Asia. Sequences available on Genbank from Chinese (2013) and Mongolian isolates (2011) show that these viruses share a different substitution at position 144 of HA (A144T) compared to the European clade 2 viruses. Careful monitoring of these three sub-groups is needed to ensure that appropriate strains are included in vaccines to protect against these groups, although at present there is no evidence to suggest that the current recommended strain (Richmond/1/07) would not provide adequate protection against either of the sub-groups circulating in Europe.

In conclusion, FC2 has continued to diverge, with the 144-group viruses predominating in the UK and the 179-group viruses in Europe, however there is a shortage of sequence data available from Europe for recent years. There appears to be three sub-groups within FC2: UK (A144V), Europe (I179V) and Asia (A144T). There is no evidence of significant antigenic drift away from the recommended vaccine strains. The recommendations to include FC2 as well as FC1 therefore still stand. The viruses have acquired further mutations in both HA and NA, therefore careful monitoring is required to identify antigenic drift at an early stage.

## 4. Materials and Methods

### 4.1. Diagnostic Testing for the Presence of EIV

Extracts from nasopharyngeal swabs were tested for EIV by the Diagnostic Laboratory Services at the AHT. One sample was assayed using an in-house nucleoprotein (NP)-ELISA as described previously [40]. The remaining samples were tested by qRT-PCR. RNA was extracted using a High Pure PCR Template Preparation kit (Roche) according to manufacturer’s instructions. Primers and probes were designed to conserved regions of segments 5 (NP) and 7 (matrix (M1)) as follows- NP forward primer: 5′-TTCTGGAGAGGTGAAAATGG-3′, reverse primer: 5′-CATAAACACAGGCAGGTAGG-3′, probe: 5′-FAM™-ACCAGAATTGCTTATGAAAGAATG-BHQ1-3′. M1 forward primer: 5′-AGATGAGTCTTCTRACCGAGGTCG-3′, reverse primer: 5′-TGCAAAARACATCTTCAAGTCTCTG-3′, probe: 5′-JOE™-TCGGCTTTGAGGGGGCCTGA-BHQ1-3′. qRT-PCR reagent mixes were set up using the SensiFAST Probe Onestep kit (Bioline: London, UK) Reactions were carried out on a StepOnePlus machine (Applied Biosystems: Warrington, UK) with cycling conditions as follows: reverse transcription at 42 °C for 10 min, an activation step at 95 °C for 2 min, then 40 cycles of denaturation at 95 °C for 5 s and primer annealing and extension at 60 °C for 30 s. Absorbance readings at 500 nm (FAM^TM^) and 560 nm (JOE^TM^) were taken following each cycle. qRT-PCR results were analysed using the StepOne^TM^ software version 2.3 (Applied Biosystems).

### 4.2. Virus Isolation in Eggs

From samples that tested positive by qRT-PCR or NP-ELISA, 100 μL of swab extract was inoculated into the allantoic cavity of two 10 day-old embryonated hens’ eggs each at neat, 10^−1^ and 10^−2^ dilutions made in sterile phosphate buffered saline containing 2% tryptone phosphate broth solution (Sigma: Gillingham, UK), 500 units/mL penicillin and 0.5 mg/ml streptomycin (Sigma: Gillingham, UK). Inoculated eggs were incubated at 34 °C for 72 h, chilled at 4 °C overnight and the allantoic fluid harvested and tested for the presence of virus by haemagglutination assay [41]. Samples testing negative by haemagglutination assay after one round in eggs were passaged up to three times in eggs, checking for growth at each step to minimise the total number of passages.

### 4.3. RNA Extraction, RT-PCR and Sequencing

RNA was extracted either directly from swab material or from allantoic fluid using a QIAampViral RNA mini kit (Qiagen: Manchester, UK) according to the manufacturer’s instructions. Amplification of the HA and NA segments was carried out by RT-PCR using Superscript II reverse transcriptase (Life Technologies: Paisley, UK) and Native Pfu DNA polymerase (Agilent: Stockport, UK) using primer designs and cycling conditions as previously described [42] to produce overlapping fragments of approximately 500 nucleotides flanked by M13 forward and reverse sequences. Sanger sequencing was carried out using BigDye Terminator Sequencing kit version 3.1 (Applied Biosystems: Warrington, UK) with M13 forward and reverse primers on an ABI PRISM 3130XL Genetic Analyser (Applied Biosystems: Warrington, UK). Raw nucleotide sequence reads were analysed and edited using Seqman 2 version 5.03 (DNAstar).

### 4.4. Sequence Analysis and Phylogenetic Trees

Nucleotide alignments were created using ClustalW multiple alignment in BioEdit version 7.0.5 (Ibis Pharmaceuticals Inc.). Phylogenetic trees were constructed using PhyML version 3 [43] and the General Time Reversible substitution model. One hundred bootstrap replicates were performed to estimate statistical significance at major branch points. In total, 189 HA sequences and 104 NA sequences were used to construct the trees.

### 4.5. Haemagglutination Inhibition (HI) Assay

Post-infection ferret antisera from AHT archives were used to conduct antigenic characterisation of untreated viruses by HI assay. The antisera were treated with periodate and heat [41] and serially-diluted across a 96-well plate. The viruses were diluted to 4 HA units and incubated with the diluted antisera at room temperature for 30 min before 1% chicken blood was added and plates incubated at 4 °C for 45 min. HI titres were recorded as the highest dilution of antisera to show full haemagglutination inhibition. Assays were carried out in duplicate and geometric mean titres calculated.

## Figures and Tables

**Table 1:** Virus isolates from outbreaks of equine influenza virus (EIV) in the UK from 2013 to 2015 including locations, detection methods and GISAID accession numbers.

Date	Location (county)	Country	Detection method	Isolate name(s)	HA acc.	NA acc.
2013						
Feb	Ayrshire	Scotland	qRT-PCR	Ayrshire/1/13	EPI492691	EPI497568
				Ayrshire/2/13	EPI838696	EPI838697
Jun	Hertfordshire	England	qRT-PCR	Hertfordshire/1/13	EPI492692	EPI497569
Jul	Northamptonshire	England	qRT-PCR	Northamptonshire/1/13	EPI492817	EPI493625
Jul	Shropshire	England	qRT-PCR	Shropshire/1/13	EPI838698	EPI838699
				Shropshire/2/13	EPI838700	EPI838701
Sep	Worcestershire	England	qRT-PCR	Worcestershire/1/13	EPI492821	EPI497570
Sep	Warwickshire	England	qRT-PCR	Warwickshire/1/13	EPI492822	EPI497571
Sep	Northamptonshire	England	qRT-PCR	Northamptonshire/3/13	EPI838702	EPI838703
				Northamptonshire/5/13	EPI838916	EPI838917
Sep	East Yorkshire	England	qRT-PCR	East Yorkshire/1/13	EPI492823	EPI497575
Sep	Lincolnshire	England	qRT-PCR	Lincolnshire/1/13	EPI838918	EPI838919
Sep	Lanarkshire	Scotland	qRT-PCR	Lanarkshire/1/13	EPI493612	EPI493613
Oct	Northamptonshire	England	qRT-PCR	Northamptonshire/7/13	EPI493616	EPI493617
Oct	Shropshire	England	qRT-PCR	Shropshire/5/13	EPI493619	EPI493620
Dec	Shropshire	England	qRT-PCR	Shropshire/8/13	EPI493622	EPI493623
2014						
Mar	North Yorkshire	England	qRT-PCR	North Yorkshire/1/14	EPI821293	EPI821294
Aug	West Lothian	Scotland	qRT-PCR	West Lothian/1/14	EPI839642	EPI839656
Aug	Bedfordshire	England	qRT-PCR	Bedfordshire/2/14	EPI821287	EPI821288
Sep	Perthshire	Scotland	qRT-PCR	Perthshire/1/14	EPI839711	EPI839712
Sep	Essex	England	qRT-PCR	Essex/1/14	EPI821295	EPI821296
Sep	Staffordshire	England	qRT-PCR	Staffordshire/1/14	EPI839713	EPI839714
Oct	South Ayrshire	Scotland	qRT-PCR	South Ayrshire/1/14	EPI821285	EPI821286
Oct	Scottish Borders	Scotland	qRT-PCR	Scottish Borders/1/14	EPI839715	EPI839716
Oct	Ayrshire	Scotland	qRT-PCR	Ayrshire/2/14	EPI821145	EPI821202
Oct	Perthshire	Scotland	qRT-PCR	Perthshire/2/14	EPI839717	EPI839718
Oct	Worcestershire	England	qRT-PCR	Worcestershire/2/14	EPI839719	EPI839720
Oct	Kent	England	qRT-PCR	Kent/1/14	EPI839721	EPI839722
Oct	West Midlands	England	NP ELISA	West Midlands/1/14	EPI839723	EPI839724
Nov	Cambridgeshire	England	qRT-PCR	Cambridgeshire/1/14	EPI821289	EPI821290
Nov	Buckinghamshire	England	qRT-PCR	Buckinghamshire/1/14	EPI651398	EPI651400
Nov	East Yorkshire	England	qRT-PCR	East Yorkshire/1/14	EPI821291	EPI821292
Nov	Gloucestershire	England	qRT-PCR	Gloucestershire/1/14	EPI821297	EPI821298
2015						
Mar	North Yorkshire	England	qRT-PCR	North Yorkshire/1/15	EPI686738	EPI686739
Apr	Leicestershire	England	qRT-PCR	Leicestershire/1/15	EPI687475	EPI694842
Jun	South Lanarkshire	Scotland	qRT-PCR	South Lanarkshire/1/15	EPI643192	EPI643193
Jul	West Midlands	England	qRT-PCR	West Midlands/1/15	EPI650051	EPI650052
Jul	Lanarkshire	Scotland	qRT-PCR	Lanarkshire/2/15	EPI650053	EPI650054
Jul	Tyne & Wear	England	qRT-PCR	Tyne & Wear/1/15	EPI650056	EPI651799
				Tyne & Wear/2/15	EPI651395	EPI650055
Jul	Scottish Borders	Scotland	qRT-PCR	Scottish Borders/3/15	EPI710985	EPI710986
Aug	Norfolk	England	qRT-PCR	Norfolk/1/15	EPI686736	EPI686737
Oct	Kent	England	qRT-PCR	Kent/1/15	EPI673530	EPI673531
Nov	East Sussex	England	qRT-PCR	East Sussex/1/15	EPI673532	EPI673533
Dec	Northamptonshire	England	qRT-PCR	Northamptonshire/2/15	EPI686740	EPI839779
				Northamptonshire/3/15	EPI839794	EPI839809
				Northamptonshire/4/15	EPI839821	EPI839822

HA acc.—Haemagglutinin GISAID accession numbers, NA acc.—Neuraminidase GISAID accession numbers, qRT-PCR—quantitative reverse transcription polymerase chain reaction, NP-ELISA – nucleoprotein enzyme linked immunosorbent assay.

**Table 2:** Haemagglutination inhibition titres of equine influenza virus strains using ferret antisera.

	Reference ferret antisera									
	New/1/93	New/2/93	Rich/1/07	Dev/1/11	ER/2/11	Ayr/1/13	SA/4/03	Dor/09	RGdS/1/12	Ky/1/14
	Am	Eu	FC2	FC2	FC2	FC2	FC1	FC1	FC1	FC1
**Reference strains**										
Newmarket/1/93	**362**	11	362	181	256	362	32	64	32	64
Newmarket/2/93	91	**512**	64	128	64	128	<8	32	8	23
**Richmond/1/07**	181	32	**256**	91	128	256	64	128	128	128
Devon/1/11	362	64	724	**512**	362	512	181	256	128	256
East Renfrewshire/2/11	256	32	724	256	**256**	512	128	256	128	256
Ayrshire/1/13	256	16	512	256	128	**724**	64	128	45	256
**South Africa/4/03**	23	8	91	64	45	181	**256**	362	256	512
Dorset/09	64	32	181	91	91	256	724	**1024**	1024	2048
Rio Grande do Sul/1/12	128	23	256	128	64	256	724	1024	**1024**	2048
Kentucky/1/14	64	16	128	128	64	128	1024	2048	2896	**2896**
**2013-15 isolates**										
Ayrshire/2/13	362	16	724	181	128	724	128	256	91	362
Northamptonshire/1/13	724	91	1024	724	362	724	128	256	181	256
Shropshire/1/13	362	32	512	362	128	256	91	256	128	256
Northamptonshire/3/13	362	64	1024	512	256	512	128	256	181	256
Shropshire/8/13	256	32	512	362	128	256	128	191	128	256
North Yorkshire/1/14	512	64	1024	512	181	512	64	362	181	256
Scottish Borders/1/14	256	64	1024	512	256	512	128	256	128	256
Perthshire/2/14	256	32	724	362	128	256	128	256	64	256
Kent/1/14	256	32	724	256	128	256	128	256	128	256
Buckinghamshire/1/14	362	32	1024	256	91	362	91	256	91	256
Gloucestershire/1/14	362	45	1024	512	181	512	128	256	128	256
South Lanarkshire/1/15	256	16	362	256	181	512	64	128	64	256
Lanarkshire/2/15	256	16	362	128	91	362	64	128	64	256
Scottish Borders/3/15	512	32	512	256	128	512	64	181	64	362
Kent/1/15	256	32	512	256	128	256	128	256	91	256
East Sussex/1/15	181	23	362	181	64	256	32	91	45	91
Northamptonshire/2/15	512	23	362	362	91	724	64	128	32	256
Northamptonshire/3/15	362	16	256	362	128	512	64	128	64	256

Geometric mean titres from duplicate assays are shown. Isolate names are indicated on the left and ordered by outbreak date. Homologous titres for reference antisera are shown in bold. Reference antisera: New/1/93—A/eq/Newmarket/1/1993, New/2/93—A/eq/Newmarket/2/1993, Rich/1/07—A/eq/Richmond/1/2007, Dev/1/11—A/eq/Devon/1/2011, ER/2/11—A/eq/East Renfrewshire/2/2011, Ayr/1/13—A/eq/Ayrshire/1/2013, SA/4/03—A/eq/South Africa/4/2003, Dor/09—A/eq/Dorset/2009, RGdS/1/12—A/eq/Rio Grande do Sul/1/2012, Ky/1/13—A/eq/Kentucky/1/2014. Am—American lineage, Eu—Eurasian lineage, FC1—Florida sub-lineage clade1, FC2 – Florida sub-lineage clade 2. OIE recommended vaccine strains are highlighted in bold.

## Supplementary Materials

The following are available online at www.mdpi.com/2076-0817/6/1/6/s1, Figure S1: Amino acid alignment comparing amino acid numbers of equine influenza virus N8 neuraminidase against H5N1 neuraminidase from 2HTY pdb structure file, Table S1: Genetic similarity between the 144- and 179-groups and the recommended FC2 vaccine strain.

## References

[1] Weis W, Brown J.H., Cusack S., Paulson J.C., Skehel J.J., Wiley D.C. (1988). Structure of the influenza virus haemagglutinin complexed with its receptor, sialic acid. Nature.

[2] Skehel J.J., Wiley D.C. (2000). Receptor binding and membrane fusion in virus entry: The influenza hemagglutinin. Annu. Rev. Biochem..

[3] Matrosovich M.N., Matrosovich T.Y., Gray T., Roberts N.A., Klenk H.D. (2004). Neuraminidase is important for the initiation of influenza virus infection in human airway epithelium. J. Virol..

[4] Halbherr S.J., Ludersdorfer T.H., Ricklin M., Locher S., Berger Rentsch M., Summerfield A., Zimmer G. (2015). Biological and protective properties of immune sera directed to influenza virus neuraminidase. J. Virol..

[5] Yamanaka T., Tsujimura K., Kondo T., Hobo S., Matsumura T. (2006). Efficacy of oseltamivir phosphate to horses inoculated with equine influenza A virus. J. Vet. Med. Sci..

[6] Yamanaka T., Bannai H., Nemoto M., Tsujimura K., Kondo T., Muranaka M., Hobo S., Minamijima Y.H., Yamada M., Matsumura T. (2012). Efficacy of a single intravenous dose of the neuraminidase inhibitor peramivir in the treatment of equine influenza. Vet. J..

[7] Waddell G.H., Teigland M.B., Sigel M.M. (1963). A new influenza virus associated with equine respiratory disease. J. Am. Vet. Med. Assoc..

[8] Bryant N.A., Rash A.S., Russell C.A., Ross J., Cooke A., Bowman S., MacRae S., Lewis N.S., Paillot R., Zanoni R. (2009). Antigenic and genetic variations in European and North American equine influenza virus strains (H3N8) isolated from 2006 to 2007. Vet. Microbiol..

[9] Woodward A.L., Rash A.S., Blinman D., Bowman S., Chambers T.M., Daly J.M., Damiani A., Joseph S., Lewis N., McCauley J.W. (2014). Development of a surveillance scheme for equine influenza in the UK and characterisation of viruses isolated in Europe, Dubai and the USA from 2010 to 2012. Vet. Microbiol..

[10] Burrows R., Denyer M., Goodrige D., Hamilton F. (1981). Field and laboratory studies of equine influenza viruses isolated in 1979. Vet. Rec..

[11] Cowled B., Ward M.P., Hamilton S., Garner G. (2009). The equine influenza epidemic in Australia: Spatial and temporal descriptive analyses of a large propagating epidemic. Prev. Vet. Med..

[12] Livesay G.J., O’Neill T., Hannant D., Yadav M.P., Mumford J.A. (1993). The outbreak of equine influenza (H3N8) in the United Kingdom in 1989: Diagnostic use of an antigen capture ELISA. Vet. Rec..

[13] Ito M., Nagai M., Hayakawa Y., Komae H., Murakami N., Yotsuya S., Asakura S., Sakoda Y., Kida H. (2008). Genetic Analyses of an H3N8 Influenza Virus Isolate, Causative Strain of the Outbreak of Equine Influenza at the Kanazawa Racecourse in Japan in 2007. J. Vet. Med. Sci..

[14] Newton J.R., Daly J.M., Spencer L., Mumford J.A. (2006). Description of the outbreak of equine influenza (H3N8) in the United Kingdom in 2003, during which recently vaccinated horses in Newmarket developed respiratory disease. Vet. Rec..

[15] Virmani N., Bera B.C., Singh B.K., Shanmugasundaram K., Gulati B.R., Barua S., Vaid R.K., Gupta A.K., Singh R.K. (2010). Equine influenza outbreak in India (2008–09): Virus isolation, sero-epidemiology and phylogenetic analysis of HA gene. Vet. Microbiol..

[16] Alves Beuttemmüller E., Woodward A., Rash A., Dos Santos Ferraz L.E., Fernandes Alfieri A., Alfieri A.A., Elton D. (2016). Characterisation of the epidemic strain of H3N8 equine influenza virus responsible for outbreaks in South America in 2012. Virol. J..

[17] Perglione C.O., Gildea S., Rimondi A., Miño S., Vissani A., Carossino M., Cullinane A., Barrandeguy M. (2016). Epidemiological and virological findings during multiple outbreaks of equine influenza in South America in 2012. Influenza Other Respir. Viruses.

[18] Yamanaka T., Niwa H., Tsujimura K., Kondo T., Matsumura T. (2008). Epidemic of equine influenza among vaccinated racehorses in Japan in 2007. J. Vet. Med. Sci..

[19] Worobey M., Han G.Z., Rambaut A. (2014). A synchronised global sweep of the internal genes of modern avian influenza virus. Nature.

[20] Lai A.C., Chambers T.M., Holland R.E., Morley P.S., Haines D.M., Townsend H.G., Barrandeguy M. (2001). Diverged evolution of recent equine-2 influenza (H3N8) viruses in the Western Hemisphere. Arch. Virol..

[21] Bernardino P.N., Mapes S.M., Corbin R., Pusterla N. (2016). Pyrosequencing as a fast and reliable tool to determine clade affiliation for equine Influenza A virus. J. Vet. Diagn. Investig..

[22] Gildea S., Quinlivan M., Arkins S., Cullinane A. (2012). The molecular epidemiology of equine influenza in Ireland from 2007 to 2010 and its international significance. Equine Vet. J..

[23] Gildea S., Fitzpatrick D.A., Cullinane A. (2013). Epidemiological and virological investigations of equine influenza outbreaks in Ireland (2010–2012). Influenza Other Respir. Viruses.

[24] Legrand L.J., Pitel P.H., Cullinane A.A., Fortier G.D., Pronost S.L. (2015). Genetic evolution of equine influenza strains isolated in France from 2005 to 2010. Equine Vet. J..

[25] Qi T., Guo W., Huang W.Q., Li H.M., Zhao L.P., Dai L.L., He N., Hao X.F., Xiang W.H. (2010). Genetic evolution of equine influenza viruses isolated in China. Arch. Virol..

[26] Yondon M., Heil G.L., Burks J.P., Zayat B., Waltzek T.B., Jamiyan B.O., McKenzie P.P., Krueger W.S., Friary J.A., Gray G.C. (2013). Isolation and characterization of H3N8 equine influenza A virus associated with the 2011 epizootic in Mongolia. Influenza Other Respir. Viruses.

[27] Binns M.M., Daly J.M., Chirnside E.D., Mumford J.A., Wood J.M., Richards C.M., Daniels R.S. (1993). Genetic and antigenic analysis of an equine influenza H 3 isolate from the 1989 epidemic. Arch. Virol..

[28] Barbic L., Madic J., Turk N., Daly J. (2009). Vaccine failure caused an outbreak of equine influenza in Croatia. Vet. Microbiol..

[29] Cullinane A., Elton D., Mumford J. (2010). Equine influenza—Surveillance and control. Influenza Other Respir. Viruses.

[30] The World Organisation for Animal Health (OIE) (2016). Expert surveillance panel on equine influenza vaccine composition—Conclusions and recommendations. Off. Int. Epizoot. Bull..

[31] GISAID EpiFlu Database (2006). Editorial: Action stations: The time for sitting on flu data is over. Nature.

[32] Collins P.J., Vachieri S.G., Haire L.F., Ogrodowicz R.W., Martin S.R., Walker P.A., Xiong X., Gamblin S.J., Skehel J.J. (2014). Recent evolution of equine influenza and the origin of canine influenza. Proc. Natl. Acad. Sci. USA.

[33] Russell R.J., Haire L.F., Stevens D.J., Collins P.J., Lin Y.P., Blackburn G.M., Hay A.J., Gamblin S.J., Skehel J.J. (2006). The structure of H5N1 avian influenza neuraminidase suggests new opportunities for drug design. Nature.

[34] The World Organisation for Animal Health (OIE) (2010). Expert surveillance panel on equine influenza vaccine composition—Conclusions and recommendations. Off. Int. Epizoot. Bull..

[35] Bryant N.A., Rash A.S., Woodward A.L., Medcalf E., Helwegen M., Wohlfender F., Cruz F., Herrmann C., Borchers K., Tiwari A. (2011). Isolation and characterisation of equine influenza viruses (H3N8) from Europe and North America from 2008 to 2009. Vet. Microbiol..

[36] Corti D., Suguitan A.L., Pinna D., Silacci C., Fernandez-Rodriguez B.M., Vanzetta F., Santos C., Luke C.J., Torres-Velez F.J., Temperton N.J. (2010). Heterosubtypic neutralizing antibodies are produced by individuals immunized with a seasonal influenza vaccine. J. Clin. Investig..

[37] Dreyfus C., Ekiert D.C., Wilson I.A. (2013). Structure of a classical broadly neutralizing stem antibody in complex with a pandemic H2 influenza virus hemagglutinin. J. Virol..

[38] Yamanaka T., Cullinane A., Gildea S., Bannai H., Nemoto M., Tsujimura K., Kondo T., Matsumura T. (2015). The potential impact of a single amino-acid substitution on the efficacy of equine influenza vaccines. Equine Vet. J..

[39] Yamanaka T., Nemoto M., Bannai H., Tsujimura K., Kondo T., Matsumura T., Gildea S., Cullinane A. (2016). Assessment of antigenic difference of equine influenza virus strains by challenge study in horses. Influenza Other Respir. Viruses.

[40] Cook R.F., Sinclair R., Mumford J.A. (1988). Detection of influenza nucleoprotein antigen in nasal secretions from horses infected with A/equine influenza (H3N8) viruses. J. Virol. Methods.

[41] OIE (2016). Chapter 2.5.7 Equine influenza. Manual of Diagnostic Tests and Vaccines for Terrestrial Animals.

[42] Rash A., Woodward A., Bryant N., McCauley J., Elton D. (2014). An efficient genome sequencing method for equine influenza [H3N8] virus reveals a new polymorphism in the PA-X protein. Virol. J..

[43] Guindon S., Dufayard J.F., Lefor V., Anisimova M., Hordijk W., Gascuel O. (2010). New algorithms and methods to estimate maximum-likelihood phylogenies: Assessing the performance of PhyML 3.0. Syst. Biol..

