# Supplementary material for: Evolution and Divergence of H3N8 Equine Influenza Viruses Circulating in the United Kingdom from 2013 to 2015
Source: Pathogens. 2017 Feb 8;6(1):6. doi: 10.3390/pathogens6010006 (PMC5371894; doi:10.3390/pathogens6010006)
Supplement: Supplementary file 1 [file pathogens-06-00006-s001.pdf]

Supplementary Materials: Evolution and Divergence of H3N8 Equine Influenza Viruses Circulating in the United Kingdom from 2013 to 2015

Adam Rash, Rachel Morton, Alana Woodward, Olivia Maes, John McCauley, Neil Bryant and Debra Elton

Figure S1. Amino acid alignment comparing amino acid numbers of equine influenza virus N8 neuraminidase against H5N1 neuraminidase from 2HTY pdb structure file.

```
                          ....|....| ....|....| ....|....| ....|....| ....|....| ....|....| ....|....| ....|....|
N1 numbering:                     10         20         30         40         50         60         70         80
N8 numbering:                     8          18         28         38         48         58         68         78
N1 (H5N1: 2HTY)           ---------- ---------- ---------- ---------- ---------- ---------- ---------- ----------
N8 (Equine: KF559336)     --MNPNQKII TIGSASLGIL IINVILHVVS IIVTVLVLNN NETGLNCKGT IIREYNETVR VEKITQWHNT SAIKYIERPP

                          ....|....| ....|....| ....|....| ....|....| ....|....| ....|....| ....|....| ....|....|
N1 numbering:                     90        100        110        120        130        140        150        160
N8 numbering:                     88         98        108        118        128        138        148        158
N1 (H5N1: 2HTY)           --VKLAGNSS LCPINGWAVY SKDNSIRIGS KGDVFVIREP FISCSHLECR TFFLTQGALL NDKHSNGTVK DRSPHRTLMS
N8 (Equine: KF559336)     NEYYMNNTEP ..EAQ.F.PF ....G..... R.H....... .V...PS... .......S.. .......... ....Y.....

                          ....|....| ....|....| ....|....| ....|....| ....|....| ....|....| ....|....| ....|....|
N1 numbering:                    169A        179        189        199        209        219        229        239
N8 numbering:                    168        178        188        198        208        218        228        238
N1 (H5N1: 2HTY)           CPVGEAPSPY NSRFESVAWS ASACHDGTSW LTIGISGPDN GAVAVLKYNG IITDTIKSWR NNILRTQESE CACVNGSCFT
N8 (Equine: KF559336)     VKI.QS.NV. QA........ .T.....KK. M...VT.... Q.I..VN.G. VPV.I.N..A GD.......S .T.IK.N.YW

                                                                                                 *
                          ....|....| ....|....| ....|....| ....|....| ....|....| ....|....| ....|....| ....|....|
N1 numbering:                    249        259        269        279        289        299        310        320
N8 numbering:                    248        258        268        278        288        298        308        318
N1 (H5N1: 2HTY)           VMTDGPSNGQ ASYKIFKMEK GKVVKSVELD APNYHYEECS CYPNAGEITC VCRDNWHGSN RPWVSFNQNL EYQIGYICSG
N8 (Equine: KF559336)     ......A.R. .K.R...AKD .R.IGQTDIS FNGG.I.... ....E.KVE. I.....T.T. ..ILVISSD. S.TV..L.A.

                                          * **                                                      *
                          ....|....| ....|....| ....|....| ....|....| ....|....| ....|....| ....|....| ....|....|
N1 numbering:                    330        341        351        361        371        381        391        402
N8 numbering:                    328        338        348        358        368        378        388        398
N1 (H5N1: 2HTY)           VFGDNPRPND G--TGSCGPV SSNGAYGVKG FSFKYGNGVW IGRTKSTNSR SGFEMIWDPN GWTETDSSFS VKQDIVAITD
N8 (Equine: KF559336)     IPT.T..GE. SQF....TSP LG.KG..... .G.RQ.TD.. A...I.RT.. ....I.KIR. ...QNSKDQI RR.V.IDDPN

                                   * ****                   *
                          ....|....|....| ....|....| ....|....| ....|....| ....|....| ....|....| ....|....| ..
N1 numbering:                     412        418        428        439        449        459        469
N8 numbering:                     408        418        428        438        448        458        468
N1 (H5N1: 2HTY)           WSGYSGSFVQ HPELTGLDCI RPCFWVELIR GRPKESTIWT SGSSISFCGV NSDTVGWSWP DGAELPFTID K-
N8 (Equine: KF559336)     ........TL PI...KKG.L V......M.. .K.E.T.... .S...VM... DHKIAS...H ...I...D.. .M
```

* - N1 structure file (2HTY) has no 301 (yellow)

* - N1 structure file (2HTY) has no 334 (cyan)

* - N1 structure file (2HTY) has no 337 or 338 (green)

* - N1 structure file (2HTY) has no 393 (magenta)

* - N1 structure file (2HTY) has 412, 412A, 412B, 412C and 412D (red)

* - N1 structure file (2HTY) has no 434 (dark green)

Table S1. Genetic similarity between the 144- and 179-groups and the recommended FC2 vaccine strain.

		Vaccine	144-Group
HA	Vaccine	-	98.4%
(nt)	179-group	98.5%	97.9%
HA	Vaccine	-	98.2%
(aa)	179-group	98.1%	97.7%
NA	Vaccine	-	98.2%
(nt)	179-group	98.3%	96.9%
NA	Vaccine	-	98.0%
(aa)	179-group	98.7%	97.2%

Percentage similarity shown. HA = haemagglutinin, NA = neuraminidase, nt = nucleotide, aa = amino acid. Recommended vaccine strain is A/eq/Richmond/1/07. A/eq/Northamptonshire/4/15 used as the most recent isolate of the 144-group, A/eq/Ayrshire/1/13 used as the most recent isolate of the 179-group.
